# Genome-wide identification and expression analysis of the polygalacturonase gene family in sweetpotato

**DOI:** 10.1186/s12870-023-04272-1

**Published:** 2023-06-03

**Authors:** Peiwen He, Jingzhen Zhang, Zunfu Lv, Peng Cui, Ximing Xu, Melvin Sidikie George, Guoquan Lu

**Affiliations:** 1 Institute of Root and Tuber Crops, The Key Laboratory for Quality Improvement of Agricultural Products of Zhejiang Province, College of Advanced Agricultural Sciences, Zhejiang A&F University, Hangzhou, 311300 China; 2 Crop Science Department, Njala University, Njala Campus. Private Mail bag, Freetown, 999127 Sierra Leone

**Keywords:** Sweetpotato, Polygalacturonase gene family, Genome-wide identification, Gene expression

## Abstract

**Background:**

Polygalacturonase (PG), a crucial enzyme involved in pectin degradation, is associated with various plants’ developmental and physiological processes such as seed germination, fruit ripening, fruit softening and plant organ abscission. However, the members of *PG* gene family in sweetpotato (*Ipomoea batatas*) have not been extensively identified.

**Results:**

In this study, there were 103 *PG* genes identified in sweetpotato genome, which were phylogenetically clustered into divergent six clades. The gene structure characteristics of each clade were basically conserved. Subsequently, we renamed these *PGs* according to their locations of the chromosomes. The investigation of collinearity between the *PGs* in sweetpotato and other four species, contained *Arabidopsis thaliana*, *Solanum lycopersicum*, *Malus domestica* and *Ziziphus jujuba*, revealed important clues about the potential evolution of the *PG* family in sweetpotato. Gene duplication analysis showed that *IbPGs* with collinearity relationships were all derived from segmental duplications, and these genes were under purifying selection. In addition, each promoter region of IbPG proteins contained *cis*-acting elements related to plant growth and development processes, environmental stress responses and hormone responses. Furthermore, the 103 *IbPGs* were differentially expressed in various tissues (leaf, stem, proximal end, distal end, root body, root stalk, initiative storage root and fibrous root) and under different abiotic stresses (salt, drought, cold, SA, MeJa and ABA treatment). *IbPG038* and *IbPG039* were down-regulated with salt, SA and MeJa treatment. According to the further investigation, we found that *IbPG006, IbPG034 and IbPG099* had different patterns under the drought and salt stress in fibrous root of sweetpotato, which provided insights into functional differences among these genes.

**Conclusion:**

A total of 103 *IbPGs* were identified and classified into six clades from sweetpotato genome. The results of RNA-Seq and qRT-PCR suggested that *IbPG006*, *IbPG034* and *IbPG099* might play a significant role in tissue specificity as well as drought and salt stress responses, which showed valuable information for further functional characterization and application of the *IbPGs*.

**Supplementary Information:**

The online version contains supplementary material available at 10.1186/s12870-023-04272-1.

## Background

The main functions of plant cell walls are the support and connection of cells, maintenance of cell expansion and the regulation of the movement of substances in and out of cells [1]. What’s more, the cell walls are not only the barrier against pathogen invasion, but also the source of signaling molecules that can regulate plant immunity [2, 3]. The plant cell wall is composed of three polysaccharides: cellulose, hemicellulose and pectin, of which pectin consists of a chain of galacturonic acid units which are linked by α-1,4 glycosidic bonds. Surrounding cellulose and hemicellulose of pectin enhances the mechanical strength of cells and intercellular adhesion, maintains the intrinsic morphology of cells, and resists the invasion of certain pathogenic microorganisms to some extent [4, 5]. Pectinase is a general term for a class of enzymes that catalyze the degradation of pectin. Polygalacturonase (PG), Pectin Methylesterase (PME), β-galactosidase (β-GAL) and Rhamnogalacturonase (RGase) are the common pectinases, and the PG is one of the most important members of pectin hydrolases. In addition, PG can be classified into endo-polygalacturonase (endo-PG), exo-polygalacturonase (exo-PG) and rhamnose-galactosidase (oligo-PG) enzymes by the different catalyzing processes [6, 7].

PG is an enzyme that plays an important role in the alteration of cell wall structure, catalyzing the cleavage of α-1, 4-polygalacturonic acid in the pectin molecule, participating in the degradation of pectin and disintegrating the cell wall structure, which is closely related to the softening of fruits [8]. *PGs* were first found in pathogenic fungi, associated with the pathogenicity and virulence of the fungus, which played a role in degrading the primordial and intercellular layers of plant cell walls that allowed better infection of plant cells [9]. As research progressed, *PGs* were also present in plants as well [10]. In transgenic apple trees (*Malus domestica*), an over-expression of *PG* gene changed the leaf shape and resulted in early leaf shedding [11]. A total of 68 *PG* gene family members have been widely reported in *A. thaliana*. The tissue differential expression analysis showed that only 43 *AtPGs* were expressed, 40 of which were expressed in flowers, 34 in roots and pods, 30 and 31 genes in leaves and stems, respectively. Besides, 75 *PGs* were identified in *Populus* which were categorized into three classes [12]. Among the 53 *PG*s of cucumber, the members of Clade A were specifically expressed during fruit development, and the majority of the members of Clade D were highly expressed in male flower buds [13]. In addition, 5 of the 54 tomato *PG* family members have been shown to be specifically or highly expressed in fruits at various developmental stages [14]. Currently, the expression levels of *CitPG2*, *CitPG29* and *CitPG34* were correlated with citrus fruitlet abscission rates and could be stimulated by ethylene or inhibited by IAA treatment, which suggested that they might be involved in the process of citrus fruitlet abscission [15]. These studies implied that the expression patterns of *PGs* were tissue specific and the different *PGs* had a wide diverse range of functions in plants. The roles of *PGs* are not restricted to plant growth processes, but also include wound responses and host-parasite interactions [16, 17]. Notably, a series of signaling processes of defense response occurred to produce PG enzyme when tomato leaves were subjected to injury stress, which in turn induced the production of oligogalacturonide (OGA) [18]. OGA acts as an endogenous plant excitation factor to stimulate different defense responses in the plant [19, 20]. Meanwhile, *PGs* were found as an early signal gene for injury located on the vascular bundle, and the expression of *PGs* induced the production of the second messenger and further activated the expression of defense genes like PPO (Polyphenol Oxidase) and PIS (Proteinase Inhibitors) in the leaf sarcomeres [21]. Although it is obvious that *PGs* contribute to the plant defense response against disease, the mode of action is still unclear. Thus, further research was required to determine whether *PGs* affect various tissues differently and how they regulate the plant defense response.

*PGs* belong to Glycosyl Hydrolase Family 28 (GH28), which proteins contain at least one domain GH28 (Pfam00295) [22–24]. It was reported that the GH28 of AT4G20050 protein was replaced by pectinase domain III (Pfam12708) in *A. thaliana*, but *AT4G20050* has been shown to have PG activity [25]. The PG proteins have their own specific conserved domains: SPNTDG (I), GDDC (II), CGPGHG (III) and RIK (IV). Domain I and II possibly constitute catalytic sites, domain III possibly involved in catalytic reactions, and domain IV possibly interact with the ionic group of the carboxylic acid moiety in the substrate [24, 26]. *PGs* are present at almost all stages of plant growth and development, and the *PG* gene families had been identified so far in a variety of species with different quantities of members. According to different classification criteria, researchers have divided *PG* gene families into various branches, the same branch of which had a similar biological function and might provide a reference during gene function analysis [27, 28]. However, the functions of a large number of new *PG* gene family members are unknown and need to be further analysed.

Sweetpotato is an important food crop which is widely grown in tropical and subtropical areas, especially in Asia. Although *PG* genes are widely identified from many plant species, its function in sweetpotato remains unclear. In this study, we conducted genome-wide identification and analysis of *PGs* in the sweetpotato genome and identified 103 *PG* family members. Then we not only performed phylogenetic analysis of the *PGs* of *A. thaliana* and *I. batatas*, but also showed detailed analysis of gene structures, conserved motifs, collinearity relationships and *cis*-acting elements of *IbPGs*. In addition, the expression level of *IbPGs* in different tissues and under different abiotic stresses were quantitatively analysed. The data from this study will provide useful information for the future study of biological functions of *PGs* in sweetpotato and other crops.

## Results

### Identification of PGs in sweetpotato and analysis of the physicochemical properties of the proteins

Using the *A. thaliana* PG proteins as the query sequences, a total of 103 *PGs* were identified in the sweetpotato genome and named *IbPG001*-*IbPG103* according to their positions on the chromosomes (Additional file 1: Figure S1 and Additional file 3: Table S1-S2). 103 IbPGs encoded 152 (IbPG061) to 1344 (IbPG042) amino acids, correspondingly. The molecular weights ranged from 16.7 kDa to 151.4 kDa and the average value was 44.7 kDa. Additionally, the isoelectric point fluctuated widely, ranging from 4.66 (IbPG064) to 10.61 (IbPG056), of which 53% was basic and 47% was acidic. Approximately 32% IbPGs were unstable with stability index values greater than 40, the rest of members were more stable. The values of gravy varied from − 0.599 (IbPG007) to 0.142 (IbPG091), showing that 88% of the members were hydrophilic proteins (The value of gravy < 0). According to the prediction of subcellular localization, IbPG042 was on the chloroplast, IbPG021, IbPG076 and IbPG090 were on the cell wall, and all of the others were on the cell membrane (Additional file 3: Table S3).

### Phylogenetic analysis and classification of IbPGs

**Fig. 1:**
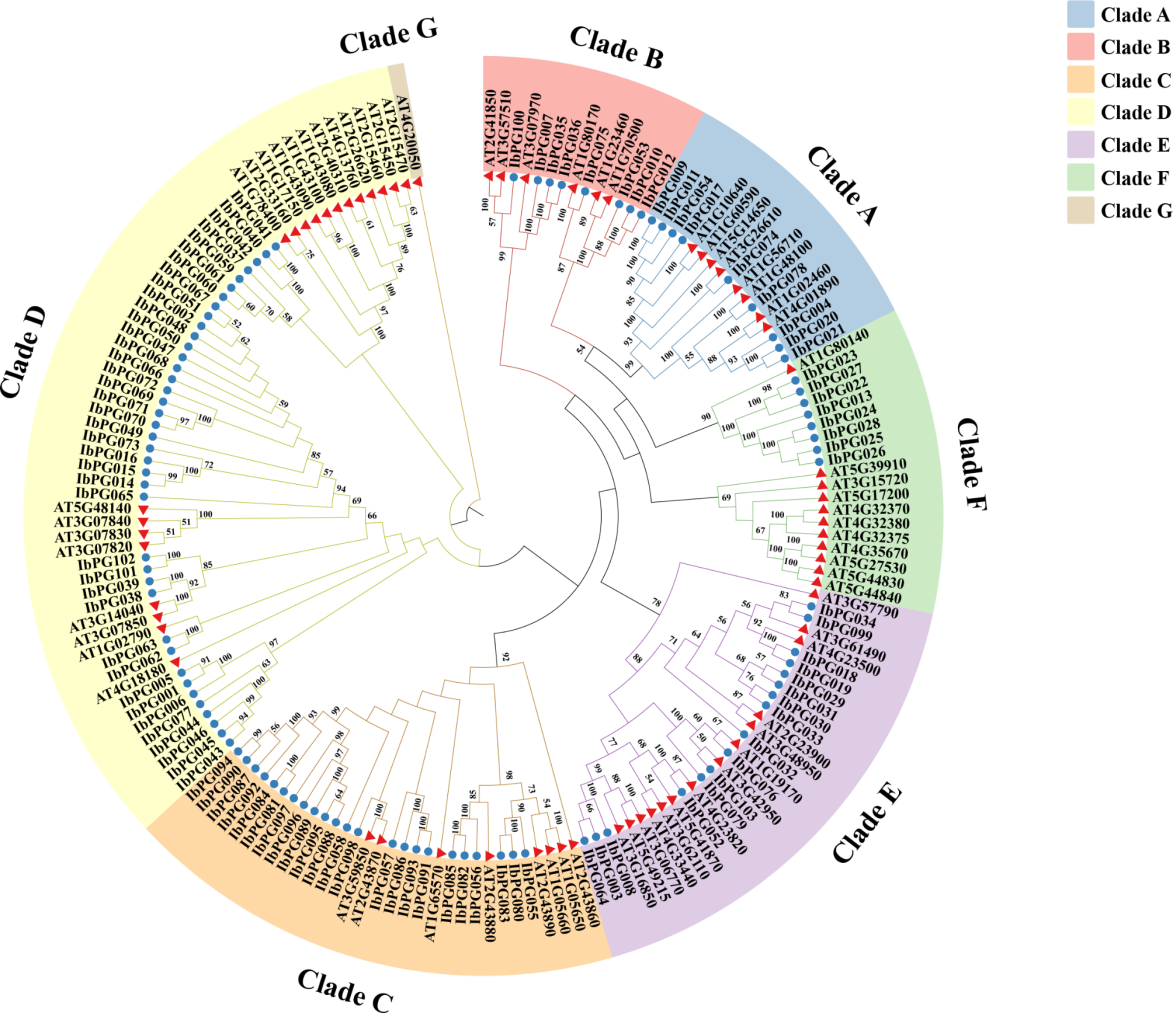
Phylogenetic analysis of *I. batatas* and *A. thaliana* constructed by MEGA6.06 software with default parameter settings. The different color blocks indicated different subclasses (Clade A-G) of the *PG* gene family. *IbPG* gene family was clustered into 6 clades, and each clade contained 9, 8, 23, 39, 16 and 8 IbPGs, respectively. The bootstrap values lower than 50 were not shown. The distance scale denoted the number of amino acid substitutions per site. The name of each branch was indicated next to the corresponding branch, the circles and triangles represented the PG proteins from *I. batatas* and *A. thaliana*, respectively

To explore the evolutionary relationships of the *PG* gene family in *I. batatas* and *A. thaliana*, an unrooted phylogenetic tree was constructed by using the protein sequences of 103 IbPGs identified and 68 *A. thaliana* PGs with the NJ method (Fig. 1). It has been reported that the PGs of *A. thaliana* were divided into seven subclasses, Clade A-G [29]. By clustering the *A. thaliana* and *I. batatas* PGs, it was discovered that the *IbPG* gene family was split into six subclasses, Clade A-F, with none IbPGs were classified into the Clade G in *A. thaliana PG* gene family. Specifically, the Clade D (39 IbPGs) contained the largest members of *I. batatas PG* family. Both Clade A and Clade B were formed of eight members, while the other clades contained 23, 16 and 9 PGs, respectively.

### Analysis of gene structure and conserved motifs

**Fig. 2:**
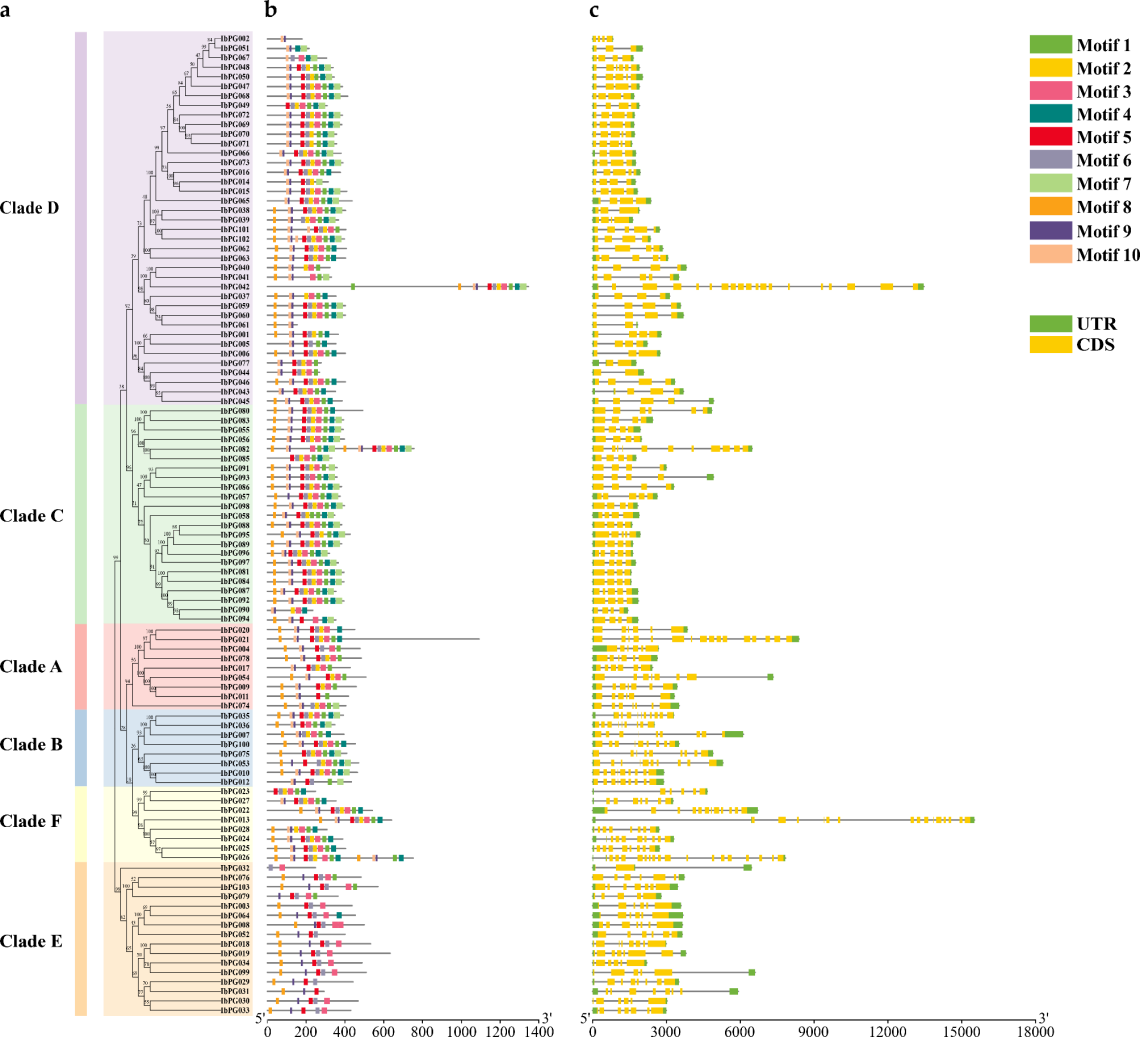
Phylogenetic relationships, architecture of conserved motifs and gene structures of the IbPG proteins from *I. batatas*. (**a**) The phylogenetic tree of IbPGs, and the blocks of different colors represented different subclasses (Clade A-F). (**b**) The distribution of conserved motifs constructed by the MEME. Boxes with different colors represented different conserved motifs (Motif 1–10). (**c**) exon/intron structure of IbPGs. Green boxes indicated the UTRs, yellow boxes represented exons, while black lines represented introns

To comprehend the structural diversity of these IbPG proteins, the exon-intron structure of each identified PG member was examined (Fig. 2). The findings showed that IbPG proteins had exons with a range of 2 (IbPG044, IbPG061, IbPG077) to 19 (IbPG032*)*. Most IbPGs in Clade A had 6–7 exons except IbPG021 with 16 exons. All IbPGs in Clade B possessed 9 or 10 exons except IbPG082 (11 exons). The members of Clade C had 4–6 exons, while the majority of genes in Clade E contained 6–8 exons except IbPG032 (2 exons). The structure of exons and introns varies between clades, but the position and length of exons and introns were relatively conserved within the same clade [13]. IbPG010 and IbPG012, IbPG015 and IbPG065, IbPG059 and IbPG060, IbPG069 and IbPG072, IbPG070 and IbPG071, IbPG081 and IbPG084 had the same gene structure (Fig. 2c).

In the investigation of the conserved domains of PG protein sequences, domain SPNTDG (I), GDDC (II), CGPGHG (III) and RIK (IV) were discovered to be present. Using the MEME website to identify and analyse the conserved motifs of 103 IbPG proteins, 10 conserved motifs were obtained, named motif 1–10 (Fig. 2b and Additional file 2: Figure S2). Motif 5, 6, 2 and 3 correspond to domain I, II, III and IV, respectively. In detail, none of the four typical conserved domains were present in IbPG002, IbPG029, IbPG031, IbPG051 and IbPG006, while 59 PG proteins possessed all of these domains. There were 11 PG proteins contained domain I, II and IV, but 6 PG proteins had only domain IV. 14 PG proteins possessed domain I and II, domain III and IV were absent in 3 PG proteins, and 5 PG proteins contained domain IV. It was similar to the research, none of the Clade E members had conserved domain III [24]. In conclusion, the IbPGs in the same clade had analogical gene structure and conserved motif compositions, which strongly implied the reliability of the phylogenetic analysis used to classify subfamilies.

### Chromosomal location, collinearity analysis, gene duplication and ***Ka/Ks*** analysis

In this study, chromosomal localization, collinearity analysis, gene duplication and *Ka/Ks* analysis of *IbPGs* were carried out to examine the mechanism of amplification and evolution of the *IbPG* gene family. By obtaining information about the chromosomal localization of *IbPGs*, a total of 103 *IbPGs* were mapped to the 14 chromosomes (Chr2-Chr15) (Additional file 1: Figure S1). One *PG* gene was found on Chr4 (*IbPG013*) and Chr15 (*IbPG103*), 2 *PGs* were distributed on Chr6 (*IbPG020, IbPG021*), 13 *PGs* was found on Chr12 and 23 *PGs* were found on Chr14.

**Fig. 3:**
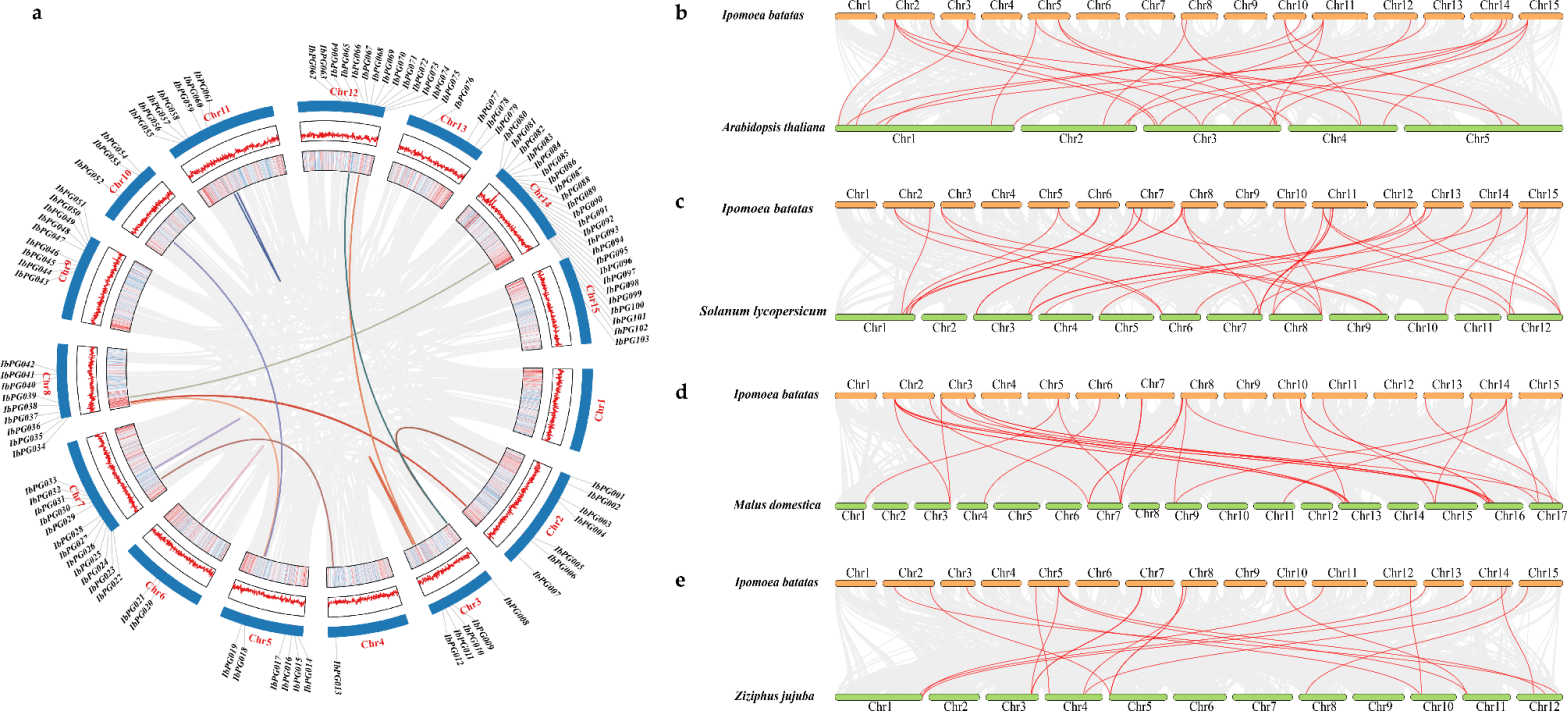
(**a**) Collinearity analysis of *IbPGs*. the lines with different colors indicated the duplicated *IbPG* gene pairs with collinearity relationships, the gray lines in the background indicated the synteny blocks of the *PGs* in the sweet potato genome. The chromosome number was indicated at the bottom of each chromosome. The line and heat map in the outer circle represent gene density on the chromosome. Collinearity analysis of *PGs* between *I. batatas* and *A. Thaliana* (**b**), *S. lycopersicum* (**c**), *M. domestica* (**d**) and *Z. jujuba* (**e**), which were constructed by TBtools. Gray lines in the background indicated the orthologous genes of *I. batatas* and other four species, while the red lines highlighted the collinearity *PG* gene pairs of *I. batatas* and other four species

In addition, collinearity analysis of the *IbPG* gene family revealed that a total of 21 *IbPGs* were involved in 11 duplication events, all of which were segmental duplications (Fig. 3a), for example, *IbPG003* on Chr2 and *IbPG008* on Chr3, *IbPG007* on Chr2 and *IbPG036* on Chr8, *IbPG010* and *IbPG012* on Chr3. Therefore, the segmental duplication events played crucial roles in the expansion of *IbPGs*.

Furthermore, a series of collinearity maps were constructed comparing sweetpotato with other four plants, *A. thaliana* (Fig. 3b), *S. lycopersicum* (Fig. 3c), *M. domestica* (Fig. 3d) and *Z. jujuba* (Fig. 3e). There were 110 pairs of collinearity relationships and the numbers of orthologous genes in *S.lycopersicum*, *A.thaliana*, *M.domestica* and *Z.jujuba* were 34, 27, 31 and 18. The largest number of collinearity relationships between *I. batatas* and *S.lycopersicum PGs* were probably due to the fact that two of them were annual herbaceous plants.

Then, the One Step MCScanX-Super Fast of TBtools software was used to calculate the Ka values, Ks values and *Ka/Ks* ratios of these identified collinearity gene pairs (Additional file 3: Table S4-S5). *Ka/Ks* is the ratio of non-synonymous substitutions (Ka) to synonymous substitutions (Ks) for 2 protein-coding genes, and the magnitude of the value can determine whether there is a selection pressure acting on this protein to code the gene [30]. Thus, the ratios reflect the evolutionary selection of the species. From the results, it can be seen that the Ks values of some collinearity gene pairs were ‘Na N’, resulting in *Ka/Ks* values were ‘Na N’. The existence of ‘Na N’ indicated the majority of synonymous mutation sites on the genes were synonymous, meaning that the sequence divergence is too great and the evolutionary distance is too long [31]. The *Ka/Ks* values of all collinearity gene pairs were less than 1, which suggested that the *IbPG* family were mainly influenced by the effect of strong purifying selection in the evolutionary processes.

### Analysis of ***cis***-acting elements in IbPGs

**Fig. 4:**
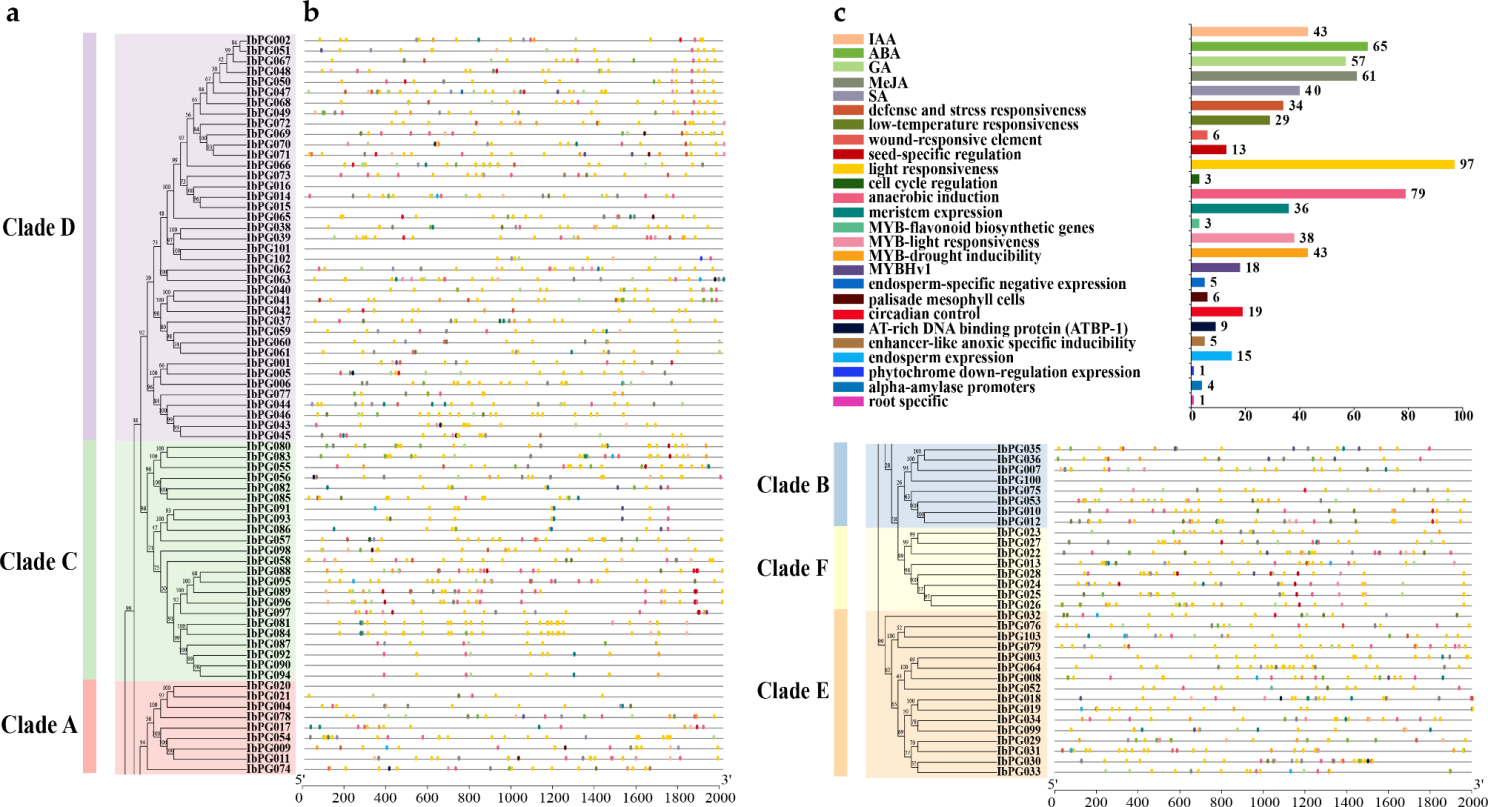
The *cis-*acting elements predication in the 2 kb sequences upstream of the IbPGs. (**a**) The phylogenetic tree of IbPGs, and the blocks of different colors represented different subclasses (Clade A-F). (**b**) The types of elements were marked by different color blocks on each line. (**c**) The column chart represented the gene numbers of *cis-*acting elements

To figure out the potential functions and regulatory mechanisms of IbPG proteins, a total of 26 *cis-*acting elements associated with different stresses and different hormone responses were identified from 2 kb region upstream of the promoters of the IbPGs using the PlantCARE online website (Fig. 4 and Additional file 3: Table S6). 97 IbPGs contained the light-responsive element and 34 IbPGs contained the defense and stress response element (TC-rich repeats). 29 IbPGs were found to possess *cis-*acting elements involved in low-temperature responsiveness (LTR). Notably, 6 IbPGs contained wound-responsive element (WUN-motif). There were five hormone response elements in the promoters of the IbPG proteins, which included the auxin-responsive element (IAA), the abscisic acid response element (ABA), the gibberellin response element (GA), the MeJA response element and the salicylic acid response element (SA). Besides, alpha-amylase promoters (RY-element) and seed-specific regulation (A-box) were also present in 4 and 13 IbPGs, respectively.

### Gene expression analysis in different tissues

**Fig. 5:**
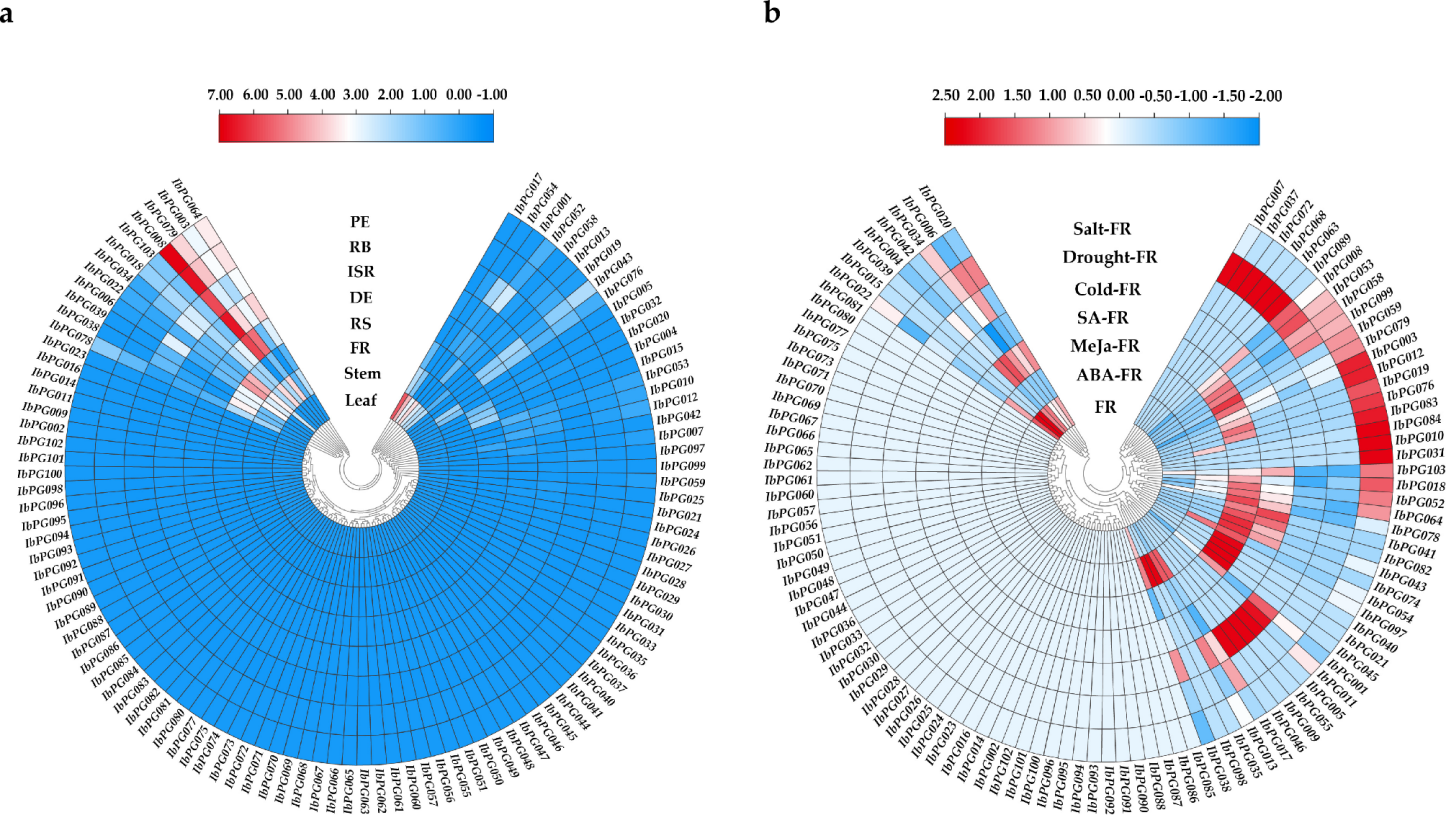
(**a**) Expression profiles of the *IbPGs* in different tissues with col scale. PE, RB, ISR, DE, RS, FR, Stem and Leaf represented different tissues of sweetpotato. (**b**) Expression profiles of the *IbPGs* in FR under different abiotic stresses and hormone treatments with row scale. All ratios were log_2_ transformed so that inductions and repressions of identical magnitude were numerically equal but opposite in sign. Red block indicated high relative expression levels and blue block indicated low relative expression levels

To investigate the expression profiles of 103 *IbPGs*, the transcriptome data of 8 tissues of the sweetpotato cultivar ‘Xushu 18’, including leaf, stem, proximal end (PE), distal end (DE), root body (RB), root stalk (RS), initiative storage root (ISR) and fibrous root (FR) were retrieved from the GEO database (PRJNA511028). According to the FPKM values (Fragments Per Kilobase of exon model per Million mapped fragments), the expression levels of the 103 genes were represented by a heatmap, as shown in Fig. 5a and Additional file 3: Table S7. The findings revealed that only one *IbPG* gene (*IbPG034*) was expressed in all tissues, but approximately 65% *IbPGs* had no detectable expression or low expression in any tissue of sweetpotato (FPKM value is less than 1). 36 *IbPGs* were expressed in at least one organ, and most of them were expressed in the root tissue (PE, DE, RB, RS, ISR and FR). The highest expression in the leaf was *IbPG007*, of which value was 3 times higher than it in the stem. The *IbPG079* displayed specifically expression in the stem, followed by *IbPG103*, *IbPG034* and *IbPG006*. Additionally, the *IbPG022* exhibited a significantly accumulation of transcripts in the FR with FPKM value larger than 10. It was noteworthy that *IbPG008* did not expressed in the leaf and FR, in contrast to other genes, it was highest expressed in the RS, DE, ISR, RB and PE with FPKM values 15, 64, 19, 60 and 45 times higher than those expressed in the stem, respectively. These results demonstrated that *IbPGs* displayed diverse expression patterns, and genes within the same subfamily also expressed differently.

### Gene expression analysis under different abiotic stress

To elucidate the possible functions of the *IbPGs*, using the published transcriptome data of the FR of ‘Xushu 18’ under drought, salt, cold, SA, MeJa, ABA inductions. The expression patterns of differentially expressed genes (DEGs) under diverse abiotic stress conditions were investigated (Fig. 5b and Additional file 3: Table S8). Overall, most *IbPGs* experienced changes in their expression levels as the findings of various abiotic stresses. Under the salt and SA treatment, the *IbPG006*, *IbPG038* and *IbPG039* from Clade D expressed down-regulated significantly. The expression of the *IbPG038* and *IbPG039* were down-regulated under the MeJa treatment. When subjected to drought, SA and MeJa treatment, *IbPG022* from Clade F showed substantially down-regulated. Meanwhile, *IbPG034* of Clade E exhibited down-regulated with the SA treatment but was up-regulated with drought and salt treatment. However, there were 2 *IbPGs* (*IbPG008* and *IbPG099*) expressed up-regulated to respond to drought treatment from the analysis of transcriptome data.

### Gene expression analysis under drought and salt stress

**Fig. 6:**
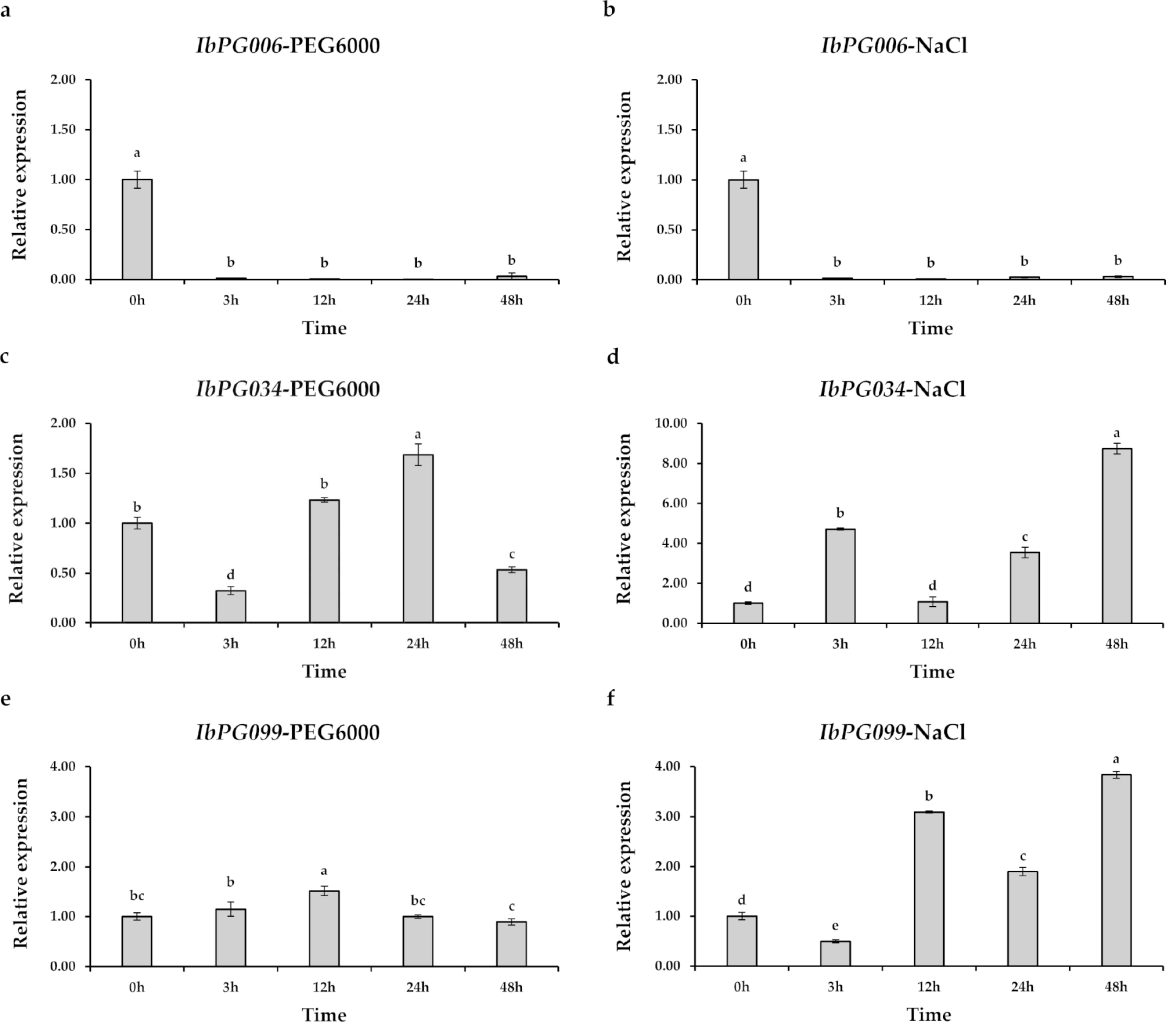
Expression profiles of the *IbPG006*, *IbPG034* and *IbPG099* under PEG6000-induced drought treatment and NaCl-induced salt treatment. (**a**) Expression patterns of *IbPG006* in response to drought stress in FR. (**b**) Expression patterns of *IbPG006* in response to salt stress in FR. (**c**) Expression patterns of *IbPG034* in response to drought stress in FR. (**d**) Expression patterns of *IbPG034* in response to salt stress in FR. (**e**) Expression patterns of *IbPG099* in response to drought stress in FR. (**f**) Expression patterns of *IbPG099* in response to salt stress in FR. The FR of ‘Xushu18’ was obtained at 0, 3, 12, 24, and 48 h after drought and salt treatment. The mean fold changes of each gene between treated and control FR were used to calculate its relative expression levels. Data are means ± SD of three biological replicates. Means denoted by the same letter do not significantly differ at p < 0.05, as determined by Duncan’s multiple range test

qRT-PCR was performed to investigate the expression dynamics of *IbPGs* over time under PEG6000-induced drought stress and NaCl-induced salt stress (Fig. 6 and Additional file 3: Table S10). The results showed that *IbPG006* was significantly down-regulated after the treatments of drought and salt at all time points, and the lowest expression levels observed for *IbPG006* corresponded to decrease of 374-fold and 114-fold, respectively. Under the drought treatment, the relative expression levels of *IbPG034* and *IbPG099* exhibited a tendency of first increasing, then decreasing, generally. *IbPG034* showed the lowest expression with 3-fold induction at 3 h, up-regulated at 12 and 24 h, and down-regulated at 48 h. *IbPG099* displayed a trend of up-regulation at 3 and 12 h and gradually down-regulated at 48 h after drought treatment. In addition, *IbPG034* was up-regulated at all periods and performed immediately increased by salt treatment at 3 h, finally expressed at a high level at 48 h. Besides, *IbPG099* displayed up-regulated at all time points except 3 h, and was most significantly expressed at 48 h. Collectively, these findings showed that different *IbPGs* had different response times to drought and salt treatment and they might express differently under abiotic stresses.

## Discussion

Plant *PGs* belong to the large Glycosyl Hydrolase Family 28 (GH28), a member of the GH superfamily in organisms, which were first identified more than 50 years ago [32]. The *PG* genes had been identified in many advanced plants recently [24]. For instance, there were 68, 46, 99, 75, 53, 62 *PGs* had been reported in *A. thaliana*, *Oryza sativa*, *Brassica oleracea*, *Populus*, *Cucumis sativus* and *Citrullus lanatus*, respectively [12, 13, 26, 28, 33]. The expression of *PG* genes played a vital role in alterations and modifications of plant cell walls. It had been demonstrated that *PGs* were expressed in various stages of plant development and different tissues [34]. Therefore, it was understandable that PG was a very important enzyme in cell wall hydrolysis. In the current study, we identified 103 *IbPGs*, which were randomly distributed on 14 chromosomes except Chr1 and clustered into six clades. It was worth mentioning that the *AT4G20050* of Clade G in *A. thaliana* did not have the homologous gene in *I. batatas*, which was similar to the results in other plants, like peach [35] and maize [36]. Moreover, IbPG proteins in the same clade shared similarity in gene structures, conserved motifs and protein physico-chemical properties. These findings were highly consistent with the PGs found in other species [6, 13]. Differences in coding regions, especially those can alter gene function, may be caused by substitution or alteration of amino acids that alter the exon-intron structures [37]. According to the analysis of the intron/exon structures, the members from Clade A, B, and F have considerably more introns, which was similar to the findings in tomato [14]. The results implied that several members of the *PG* gene family had diverged in gene structures and conserved motifs during the evolutionary process of plants, generating numerous clades with various sequence structural characteristics. Some studies demonstrated that domains I and II may constitute catalytic sites, while domain III may take part in the reaction, and domain IV may interact with the ionic groups of the carboxylic acid groups in the substrate [38]. Most IbPG proteins in our analysis possessed domain I and II, suggesting that these proteins may still be catalytically active but lost the ability to engage with the substrate that contain the ionic groups of the carboxylic acid groups. The members of Clade E were deficient in the domain III, and the similar results were observed in *B. oleracea* [33]. According to speculation, the members of Clade A and B include endo-PGs, the members of Clade C and D contain exo-PGs, Clade E members contain rhamno PGs, and Clade F members are neither exo-PGs nor endo-PGs, the variations in pectin components that each clade catalyzed may be shown by the evolutionary distinctions because of different types of PG enzyme have different substrates and products.

The chromosome localization analysis of the *IbPGs* showed that all of the members were randomly distributed on 14 chromosomes and there were 15 gene clusters. As is known to all, segmental duplication, tandem duplication, and transposition events are the main causes of gene family expansion. Segmental duplication events of homologous genes are typically referred to those occur in distant regions, whereas tandem duplication events are those occur in close regions [39]. In our study, the 11 duplicated pairs of *I. batatas* genome belonged to segmental duplication, which implied that segmental duplications were the main mode accounting for the expansion of *IbPG* gene family. At the same time, Clade E and B were the primary clades for the segmental repeat occurrences, demonstrating that different clades of *IbPGs* had various expansion modes. What’s more, the study revealed that the *Ka/Ks* ratio of the 11 *PG* gene pairs of segmental duplications was less than 1, which suggested that it was the purifying selection that lead to the duplication of the *IbPGs*, and the related IbPG proteins were thought to be largely conserved [40, 41]. The amount of evidence demonstrated that variations in promoter regions were typically connected with differences in gene activities [42, 43]. Gene expression was significantly regulated by *cis-*elements found in the promoter regions of genes during periods of growth and environmental change [44, 45]. A total of 26 *cis-*acting elements identified in *IbPG* gene family were primarily categorized into plant growth and development processes, environmental stress responses and hormone responses. Each IbPG protein contained at least one *cis-*acting element of these types, which indicated that most of PG proteins could react to a variety of abiotic stresses. Salicylic acid has been proved to be a key phytohormone that controlled both the systemic acquired disease resistance and local disease resistance mechanisms in plants [46]. We discovered that promoters of 40 IbPGs contained the *cis-*acting elements involved in salicylic acid responsiveness, suggesting that it may play a significant role in regulating the growth and development of sweetpotato, or participate in the disease resistance process. In particular, the promoter regions were connected to wound-responsive element in six proteins (IbPG001, IbPG005, IbPG031, IbPG035, IbPG036 and IbPG044), which would be a crucial point in the next study.

Gene expression profiles provided essential clues for expressing gene function [47]. To confirm the expression patterns of *IbPGs*, we further used the transcriptome data and qRT-PCR results of different tissues and diverse abiotic stresses in sweetpotato. The findings showed that the *IbPGs* were mainly expressed in the root tissue, indicating that they may play necessary functions throughout vegetative growth. The expression level of *SlPG70* peaked at the mature stage, implying that the collinear gene (*IbPG022*) might have the similar functions [14]. Members of several clades showed varying gene expression patterns in responding to different stresses [48–50]. The members from Clade D were more sensitive to different abiotic stresses than members from other clades. For instance, *IbPG038* and *IbPG039* were significantly down-regulated to response to salt, SA and MeJa treatment. In the previous study, The *Md78* might have a close functional connection with *AT1G02790*, which was a differentially expressed gene in pollen and displayed high expression levels under hypoxic stress [51–53]. Thus, it is reasonable to presume that *IbPG038* which possesses a collinearity relationship with *Md78* also has these similar functions. Collectively, the findings revealed that most *IbPGs* were down-regulated to response to various abiotic stresses. For example, *IbPG006* displayed a dramatic down-regulation after drought and salt stress, *IbPG034* and *IbPG099* also exhibited down-regulated with drought treatment at 48 h. However, we found that *IbPG034* and *IbPG099* were significantly up-regulated under the salt stress. In short, all evidence indicate that *IbPG* genes play an important role in regulating stress response in plants.

## Conclusions

In summary, 103 *PGs* were identified from sweetpotato genome, and a series of comprehensive bioinformatic analyses were performed. Phylogenetic analysis showed that these IbPG proteins were clustered into six clades of which gene structures were highly conserved in each of the clades, reflecting their functional conservation. Most *PGs* originated from fragment replication and were influenced by purification selection. In addition, promoter region of IbPG proteins contained *cis*-acting elements related to plant growth and development processes, environmental stress responses and hormone responses. Finally, from the gene expression pattern analysis, it could be seen that the most *IbPGs* were expressed in the root tissue and the expression levels of *IbPGs* were different under the drought and salt stress, which could lead to some fresh ideas for our upcoming investigations. In summary, these data provided valuable information for future functional investigations of this gene family.

## Methods

**Identification of*PG*family genes in sweetpotato genome**.

In order to identify all potential members of *PG* family in sweetpotato, the genome sequence data and gene annotation information files of *A. thaliana* and *I. batatas* were download from the TAIR database (http://www.arabidopsis.org/) and Ipomoea Genome Hub (http://sweetpotato.com/), separately. 68 PG protein sequences of *A. thaliana* were used as seed sequences to search the *I. batatas* genome with BLASTP, the E value (≤ 1e^-5^) and identity match (≥ 50%) were used as thresholds for the TBtools software [32, 54]. The Pfam database (https://pfam.xfam.org/) provided the Hidden Markov Model (HMM) profiles for the Glycosyl hydrolases family 28 (PF00295) domain, which were utilized as canonical domains to perform the HMM searching for sequence homologs using HMMER 3.3.1 (http://hmmer.org/download.html), with an E-value (≤ 1e^-5^). The potential *PGs* identified in BLASTP and Hmmer searches were screened, integrated, and then uploaded to the CDD website of NCBI for the further identification and analysis (https://www.ncbi.nlm.nih.gov/cdd/).

**Phylogenetic analysis and classification of*IbPG*gene family**.

The multiple sequence alignment of PG proteins were analysed by the Muscle program of the MEGA6.06 software with default parameter settings [55]. Then the generated multiple comparison files were imported into MEGA6.06 software to construct phylogenetic relationships between *I. batatas* and *A. thaliana* using the Neighbor-Joining (NJ) method, with the following parameters: 1000 bootstrap method, Jones-Taylor-Thornton (JTT) model, Pairwise deletion [56]. Finally, based on the previous study [6], the constructed phylogenetic tree was classified, annotated and embellished by the online tool of Chiplot (https://www.chiplot.online/).

### Chromosomal location and protein physical and chemical properties analysis

To determine and plot the chromosomal positions of the *IbPGs*, the Gene Location Visualise of TBtools software was used according to genome annotation data. The detected *PGs* were given the names *IbPG001* to *IbPG103* based on the gene localizations. The ExPASy online database ProtParam tool (http://www.expasy.org/protparam/) was used to predict and analyse the amino acid number, molecular weight (MW), isoelectric point (*pI*), instability index, aliphatic index and gravy of PG proteins [57]. The subcellular localizations of IbPGs were predicted by the webserver of Cell-PLoc 2.0 (http://www.csbio.sjtu.edu.cn/bioinf/Cell-PLoc-2/).

### Analysis of gene structure and conserved motifs

The gene structure of IbPG proteins were analysed and visualised by using Graphics of TBtools software [54]. The MEME online program (http://memesuite.org/tools/meme) was used to discover the conserved motifs of the IbPGs which were identified before, and the maximum number of bases was set to 10 in the program settings, the rest of the parameters were left as default.

### Analysis of collinearity relationship

Using the One Step MCScanX-Super Fast and File Transformat for MicroSynteny Viewer of TBtools software, the downloaded genome sequence files and genome structure annotation information files were analysed to obtain collinearity information among the *IbPGs*. Then all the output files were imported into Advanced Circos of TBtools software to obtain a visualisation of the collinearity relationship between the *IbPG* family members. Furthermore, the genome sequence files and genome structure annotation information files of *A. thaliana*, *S. lycopersicum*, *M. domestica* and *Z. jujuba* were downloaded from the TAIR website (http://www.arabidopsis.org/) and the NCBI website (https://www.ncbi.nlm.nih.gov/genome/), respectively. Then the homologous *PGs* between these four plants and sweetpotato were analysed separately using One Step MCScanX-Super Fast of TBtools software. Finally, the Dual Systeny Plot of TBtools software was used to visualise and graph the homology information.

The nonsynonymous substitution rates (Ka) and synonymous substitution rates (Ks) across paralog pairs inside *IbPGs* were computed by The *Ka/Ks* Calculator of the TBtools software.

### Analysis of ***cis***-acting elements in promoter regions of IbPG proteins

The 2 kb sequences upstream of the start codon of IbPGs were extracted and obtained by using the sequence toolkit of TBtools software, then the files were submitted to the PlantCARE website (http://bioinformatics.psb.ugent.be/webtools/plantcare/html/). After filtration and simplification, the predicted *cis*-acting component information was visualised using TBtools software.

### Analysis of gene expression of ***IbPGs***

Transcriptome data for eight different tissues of sweetpotato were obtained from previous studies with project ID PRJNA511028 in NCBI (https://www.ncbi.nlm.nih.gov/sra/), including leaf, stem, PE, DE, RB, RS, ISR and FR. In addition, transcriptome data for salt, drought, cold, SA, MeJa and ABA were described with project ID PRJNA511028 (https://www.ncbi.nlm.nih.gov/sra/). FPKM (fragments per kilobase of exon per million fragments mapped) was used to represent the gene expression level [58]. TBtools software was used to produce the heat maps while the DSEeq2 R package was used to analyse the differential expression of *IbPGs*.

### Plant materials and stress treatments

The seedlings of sweetpotato cultivar (Xushu 18) were collected from the College of Zhejiang A&F University, China. The uniform seedlings were grown in the Hoagland solution at 26 °C under a photoperiod of 16 h light/8 h dark for three days. To evaluate the expression patterns under the drought and salt stress, the seedlings were treated in the solution containing 0.2 mol/L NaCl and 20% PEG6000 mass/volume fraction, respectively. The fibrous roots were collected after 0, 3, 12, 24 and 48 h, which were immediately put in liquid nitrogen and then put at -80 °C for storage.

### Quantitative real-time PCR analysis

The expression patterns in response to abiotic stresses (PEG6000-induced drought stress and NaCl-induced salt stress) were examined in order to evaluate the role of three *IbPGs* by using qRT-PCR. All the primers used in this study were designed using the NCBI (https://www.ncbi.nlm.nih.gov/tools/primer-blast/) and listed in Table S9. Total RNA was extracted from the frozen samples by using SteadyPure Plant RNA Extraction Kit (Accurate Biotechnology, Hunan, China.) in accordance with the manufacturer’s instructions to validate the RNA-seq data. Reverse transcription was performed on 1 µg of RNA from each sample using the Evo M-MLV RT Mix Kit with gDNA Clean for qPCR (Accurate Biotechnology, Hunan, China). The qRT-PCR assay was conducted by a CFX Connect Real-Time System (Bio-Rad, Veenendaal, UT, USA) using the SYBR Green Premix Pro Taq HS qPCR Kit (Accurate Biotechnology, Hunan, China). The *β-Actin* gene (GenBank, AY905538) was used as an internal reference to evaluate the relative gene expression level. Three replicates of the tests were carried out, and the data were computed using the 2^-ΔΔCT^ method [59]. We analysed the data and compared the means using LS at a 0.01 level of significance.

## Electronic supplementary material

Below is the link to the electronic supplementary material.

Supplementary Material 1

Supplementary Material 2

Supplementary Material 3

## Data Availability

All the supporting data are included within the article and its additional files. The genome sequences of the sweetpotato, including the predicted gene model annotation for this study, can be found in Ipomoea Genome Hub (http://sweetpotato.com/). The RNA-Seq data used and analyzed during this study can be acquired from SRA of NCBI (PRJNA511028).

## Abbreviations

ABA: Abscisic acid
DEGs: differentially expressed genes
FPKM: Fragments Per Kilobase of exon model per Million mapped fragments
Gravy: Grand average of hydropathicity
HMM: Hidden markov mode
*IbPG*: *Ipomoea batatas* PG
MEME: Multiple EM for motif elicitation
PG: Polygalacturonase
PIS: Proteinase inhibitors
PME: Pectin methylesterase
PPO: Polyphenol Oxidase
qRT-PCR: Quantitative real-time PCR
RGase: Rhamnogalacturonase
β-GAL: β-galactosidase.
