# Supplementary material for: Genome-wide identification and expression analysis of the polygalacturonase gene family in sweetpotato
Source: BMC Plant Biol. 2023 Jun 3;23:300. doi: 10.1186/s12870-023-04272-1 (PMC10239142; doi:10.1186/s12870-023-04272-1)
Supplement: Supplementary file 1 — Supplementary Material 1 [file 12870_2023_4272_MOESM1_ESM.pdf]

Name	Sequence	Logo
**motif 1**	**ZBIVMSBVKNPIIIDQNYCPN**	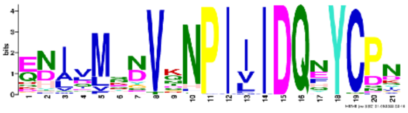
**motif 2**	**NLWIENVTCGPGHGISIGSLG**	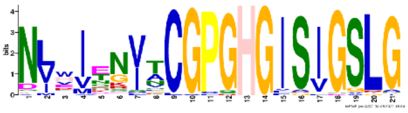
**motif 3**	**EEGVSNVTVKBCTFIGTQNGV RIKTWAGS**	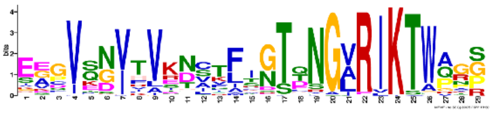
**motif 4**	**VKISNVTYKBIKGTSATEVAVI LDCS**	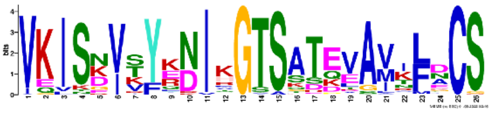
**motif 5**	**IQNVTISAPGDSPNTDGIHIQ**	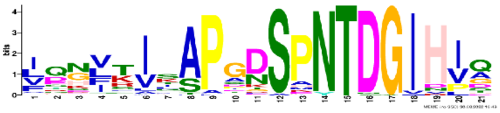
**motif 6**	**VTITBSVIGTGDDCISIGDGT**	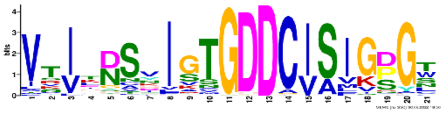
**motif 7**	**VPCEGIEJGDINLTFKGGPAK ASCSNAKGTLTG**	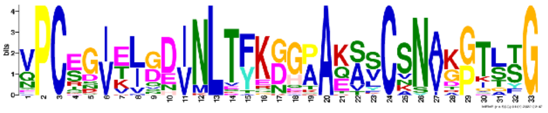
**motif 8**	**LDFGAKGDGKTDDTKAF**	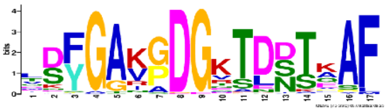
**motif 9**	**GTJDGQGAAWW**	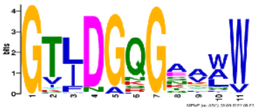
**motif 10**	**EWJTFRYVDGLTVSG**	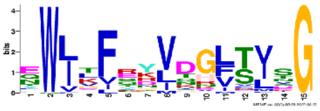

Figure S2. Sequence logo of conserved motifs found in *IbPG* gene family. Sequence logo was created by MEME online program. The X-axis showed the conserved sequences of the motifs and the conservation of residues were indicated by the height of the letters. The Y-axis represented the conservation of the amino acid.
